# Correspondence: Reply to ‘Chimpanzee helping is real, not a byproduct’

**DOI:** 10.1038/s41467-017-02328-z

**Published:** 2018-02-12

**Authors:** Keith Jensen, Claudio Tennie, Josep Call

**Affiliations:** 1 Faculty of Biology, Medicine and Health, University of Manchester, Coupland 1 Building, Coupland Street, Oxford Road, Manchester, M13 9PL UK; 2 Department for Early Prehistory and Quaternary Ecology, The University of Tübingen, Burgsteige 11, 72070 Tübingen, Germany; 3 School of Psychology and Neuroscience, University of St Andrews, Fife, St Andrews, KY16 9JU UK

## Introduction

In their response to our work^1^, the authors of the Correspondence raise some valuable points and highlight some of the confusion surrounding the nature of prosociality in nonhuman animals. They ask whether chimpanzees initially understood our task, whether recipients could signal their need, and questioned whether the stimulus-enhancement hypothesis can explain the results of our and other studies. We address each of these points in turn, and show that current evidence does not clearly support claims of chimpanzee helping.

First, the Correspondence suggests that chimpanzees failed to understand the task. As we stated in our original paper, it is possible that this was the case in experiment 1 but this criticism does not apply to experiment 2. Concerning experiment 1, there was no a priori reason to believe that chimpanzees could not learn the basic contingencies of the task by observing the consequences of their actions on others. Their failure to learn may therefore have been due to a lack of motivation rather than lack of comprehension. As in prior helping studies, actors clearly understood the task after training. Furthermore, in the training prior to testing in experiment 2, NO-GO actors released the peg in most of the initial trials of training. In other words, when there was no partner present and the actors could go from the peg room to the food box room, NO-GO actors were more likely, rather than less likely, to release the peg. The additional training suggested in the Correspondence is a completely novel action for chimpanzees; we are not aware of any study that has attempted something similar in any context. However, because this proposed action is different from any that they use in the test, it is not at all clear what chimpanzees would have learned, and it would detract from the primary purpose of the study.

Second, the Correspondence argues for the importance of signalling in eliciting prosocial behaviour. They provide a meta-analysis of helping studies showing the importance of signalling. However, they overlook the so-called sharing literature, and do not acknowledge that signalling can act as a form of harassment or even stimulus enhancement. For instance, in Melis et al.^2^, signalling and instrumental action were not disambiguated, even though the latter could also lead to stimulus enhancement. Concerning their critiques of our original study^1^, the distance between signaller and actor in was identical to Melis et al.^2^ In experiment 2, recipients did signal (as opposed to just acted on the apparatus, a distinction that was not made in a previous helping study^2^) in 50 trials in the GO group and in only six in the NO-GO group; see Table 1. This makes sense because recipients in the NO-GO group could get food without the peg being released. Signalling did not have an effect on peg releases in the NO-GO group (exact McNemar test, *N*_signal_ = 6; *N*_no-signal_ = 54; *P* = 0.581), and had a negative effect on releases in the GO group (McNemar test, *N*_signal_ = 22; *N*_no-signal_ = 50; *P* < 0.001). Thus, actors released less when they received signals from the recipient in the condition in which releasing would have had a prosocial effect. Further to the signalling hypothesis, we also re-coded the behaviour of actors from Melis, et al.^2^ and found that actors were more likely to approach the peg when the recipient was active (i.e., produced instrumental actions on the apparatus or signalled) rather than passive (Wilcoxon matched-pairs exact test: *N* = 14 (2 ties), *T* + = 70.50, *P* = 0.010; see Supplementary Table 1). However, once at the peg, there was no difference between how often the peg was actually released for passive or active recipients (*N* = 10 (4 ties), *T* + = 17.00, *P* = 0.219). In other words, recipient behaviour attracted the actor to the peg, but did not influence the act of helping. This is challenging for the signalling hypothesis but is consistent with the stimulus enhancement hypothesis. Related to the idea that the goal of the actor should be to help the recipient, the food box in our experiment was transparent, unlike what is claimed by the Correspondence; furthermore, in experiment 2, the actors had prior experience getting food from it.

**Table 1:** Recipient signalling from Tennie et al.^1^

Actor releases peg	Recipient signalling			Recipient not signalling		
	Mean	Lower bound	Upper bound	Mean	Lower bound	Upper bound
NO-GO	0.17	−0.26	0.6	0.15	0.05	0.25
GO	0.09	-0.04	0.22	0.06	0.01	0.13

Proportion of trials in which the actor released the peg when the recipient signalled (vocalising, stamping, raspberry, clapping) or did not signal for the GO and NO-GO actors in experiment 2 of Tennie et al.^1^ There were 60 NO-GO trials and 72 GO trials. A second experimenter blind to the study design coded 25% of these trials and reliability was perfect (Cohen’s kappa = 1.0). Values are shown as mean and 95% CI (lower/upper bound)

Third, the Correspondence questions the validity of the stimulus enhancement hypothesis based on the predictions derived from it: (1) no difference between GO and NO-GO and (2) a difference between test and control. However, with regard to the first prediction, NO-GO actors demonstrably understood the task by experiment 2 and the lack of a difference between the GO and NO-GO conditions is consistent with the stimulus enhancement hypothesis. With regard to the second prediction, the argument presented in the Correspondence is partially correct—a simple reading of the stimulus enhancement hypothesis would predict a difference between the test conditions and the social control. However, this hypothesis is in part an association account: chimpanzees habituated to the unreinforced stimulus. That is, the actors learned that the stimulus—the shaking food box—was not reinforced when the door leading to the room containing it was closed (as in the test and social control), so they stopped engaging with the box when there was no personal benefit to doing so. The lack of difference was thus likely due to a floor effect. Regardless, the difference between test and social control does not make the signalling hypothesis true. To do so, the Correspondence would still have to explain why chimpanzees at the peg showed no differential prosocial behaviour towards the recipient in Melis et al.^2^, why once chimpanzees were trained on the apparatus in Tennie et al.^1^ they did not release the peg at a high rate in the GO group (especially since they were doing so almost 100% of the time in the knowledge probe) and not at all in the NO-GO group (again, as in the respective knowledge probe), or why they did not release more in the GO group in the test than under the social control condition. At this point chimpanzees clearly understood what happened to the food box when they released the peg, but they were indifferent.

The central claim of our recent paper^1^ was not that the stimulus enhancement can explain everything, but that it can explain a lot. Our key point is that due to 'association blindness'^3^, alternative explanations are often overlooked in purported demonstrations of human-like motivations in non-human primates. Future work should not be blind to alternatives such as stimulus enhancement. Converging lines of evidence do not converge if they measure different things: harassment plus carry-over training effects plus stimulus enhancement does not equal prosocial motivation. We have to look beyond superficial behaviours to determine their underlying causes.

## Electronic supplementary material

Supplementary Information
