# Supplementary material for: Correspondence: Reply to ‘Chimpanzee helping is real, not a byproduct’
Source: Nat Commun. 2018 Feb 12;9:616. doi: 10.1038/s41467-017-02328-z (PMC5809583; doi:10.1038/s41467-017-02328-z)
Supplement: Supplementary file 1 — Supplementary Information [file 41467_2017_2328_MOESM1_ESM.pdf]

Supplementary Information

Supplementary Table 1. Actor behaviour from Melis et al. (2011).

	recipient passive			recipient active		
actor behaviour	mean	lower bound	upper bound	mean	lower bound	upper bound
present at peg	.35	.27	.42	.56	.43	.69
releases peg	.80	.69	.91	.79	.64	.94

Proportion of trials in which the actor was present at the peg when the recipient was passive and active, and of these trials, the proportion in which the actor released the peg once there. We coded 258 valid test trials from Melis et al. (2011) for whether the actor was at the peg at any point during the 60s trial, producing a binary (yes/no) measure. A second experimenter coded 25% of these trials and inter-observer reliability was excellent (Cohen’s kappa = 0.965). Values are shown as mean and 95% CI (lower/upper bound).
